# Field Efficacy of an Attenuated Infectious Bronchitis Variant 2 Virus Vaccine in Commercial Broiler Chickens

**DOI:** 10.3390/vetsci5020049

**Published:** 2018-05-09

**Authors:** Mohamed A. Elhady, Ahmed Ali, Walid H. Kilany, Wael K. Elfeil, Hytham Ibrahim, Ahmed Nabil, Ahmed Samir, Magdy El Sayed

**Affiliations:** 1 Department of Toxicology and Forensic Medicine, Faculty of Veterinary Medicine, Cairo University, Giza 12211, Egypt; 2 Poultry Diseases Department, Faculty of Veterinary Medicine, Beni-Suef University, Beni-Suef 65211, Egypt; ahmed.ali1@vet.bsu.edu.eg; 3 Reference Laboratory for Veterinary Quality Control on Poultry Production (NLQP), Animal Health Research Institute, P.O. Box 264, Dokki, Giza 12618, Egypt; walidhamdy78@yahoo.com; 4 Avian and Rabbit Medicine Department, Faculty of Veterinary Medicine, Suez Canal University, Ismailia 41522, Egypt; 5 Poultry Disease Department, Faculty of Veterinary Medicine, Aswan University, Aswan 81528, Egypt; haithmhmam2020@gmail.com; 6 Middle East for Veterinary Vaccine Company, Second Industrial Area, El-Salihya El-Gededa, El-Sharkia 44671, Egypt; ahmed.nabil@gmail.com (A.N.); magdy.elsayed@gmail.com (M.E.S.); 7 Microbiology Department, Faculty of Veterinary Medicine, Cairo University, Giza 12211, Egypt; ahmedsamir121@hotmail.com

**Keywords:** IBV, attenuated vaccine, infectious bronchitis virus, Middle East, variant 2

## Abstract

Egyptian poultry suffer from frequent respiratory disease outbreaks associated with Infectious Bronchitis Virus (IBV) variant 2 strains (Egy/VarII). Different vaccination programs using imported vaccines have failed to protect the flocks from field challenge. Recent studies confirmed a successful protection using homologous strains as live attenuated vaccines. In this study, a newly developed live attenuated IB-VAR2 vaccine representing the GI-23 Middle East IBV lineage was evaluated in day-old commercial broilers in an IBV-endemic area. A commercial broiler flock was vaccinated with the IB-VAR2 vaccine at day-old age followed by IB-H120 at day 16. The vaccinated flock was monitored on a weekly basis till the slaughter age. The health status and growth performance were monitored, and selected viral pathogen real-time RT-PCR (rRT-PCR) detection was conducted on a weekly basis. Finally, the flock was compared to a nearby farm with only the classical IB-H120 vaccination program. Results showed that the IB-VAR2 vaccine was tolerable in day-old broiler chicks. The IBV virus rRT-PCR detection was limited to the trachea as compared to its nephropathogenic parent virus. Respiratory disease problems and high mortalities were reported in the IB-H120-only vaccinated flock. An exposure to a wild-type Egy/VarII strain was confirmed in both flocks as indicated by partial IBV S1 gene sequence. Even though the IB-VAR2-vaccinated flock performance was better than the flock that received only IB-H120, the IBV ELISA (enzyme-linked immunosorbent assay) and log2 Haemagglutination inhibition (HI) antibody mean titers remained high (3128 ± 2713 and ≥9 log2, respectively) until the 28th day of age. The current study demonstrates the safety and effectiveness of IB-VAR2 as a live attenuated vaccine in day-old commercial broilers. Also, the combination of IB-VAR2 and classical IBV vaccines confers a broader protective immune response against IBV in endemic areas.

## 1. Introduction

Infectious bronchitis virus (IBV) is a single-stranded enveloped RNA virus that belongs to the genus *Gammacoronavirus*, family Coronaviridae [1]. The virus causes a clinical disease characterized by coughing, sneezing, tracheal rales, and watery eyes. In layer and breeder chickens, the disease is associated with both quantity and quality deterioration of egg production. Lesions in infected birds include the degeneration of renal and ciliated respiratory epithelia [2].

In Egypt, Massachusetts D3128, D274, D08880, 793B, variant 1 and 2 strains [3,4], and IS/1494/06 nephropathic IBV strain are frequently isolated from poultry [5]. Studies classified recent IBV strains into Egyptian variant 1 (Egy/VarI) and Egyptian variant 2 (Egy/VarII) based on the S1 hypervariable region 3 sequence analysis [4,6]. Currently, the IBV variant 2 is the most predominant serotype in Egypt, causing massive losses in broiler, layer, and breeder sectors [7,8]. A universal phylogeny-based classification system recently suggested that the Egyptian IBV strains belong to a unique cluster confined to the Middle East region, designated as genotype-I; lineage-23 (GI-23 lineage) [9]. The fact that many IBV serotypes and/or genotypes are cocirculating worldwide has made it difficult to control the virus. Protection studies indicated that using a homologous strain vaccine can afford better protection against IBV challenge [10,11,12]. Also, attempts to broaden the protective efficacy of IBV vaccines through experimental combinations of live IBV vaccines presented a successful strategy to protect chicken against heterologous virulent IBV strains [13]. A recent study attributed this success to the levels of cellular and local immune responses at the tracheal mucosa, which were higher when combining different live IBV vaccines in vaccination programs [14]. In this study, field safety and effectiveness of the newly developed IB-VAR2^®^ vaccine followed by a classical IB-H120 vaccine administered at 16 days of age were evaluated in day-old chicks under normal field circumstances.

## 2. Materials and Methods

### 2.1. Vaccines

The ME VAC IB VAR2^®^ employed is an IBV vaccine containing the variant 2 IBV strain Eg/1212B/2012, Genbank accession no.: JQ839287 (Middle East for Veterinary Vaccines “ME VAC”, Egypt). The classical IBV vaccine is a commercial live attenuated H120 strain (Nobilis IB H120; Intervet, Boxmeer, The Netherland). All the vaccines were given at the manufacturer’s recommended doses.

### 2.2. Study Location

The study location was selected as being a high-density area for poultry production with recent history of frequent IBV outbreaks. A naturally ventilated commercial broiler chicken farm stocked with 7600 Ross broiler chicks at El-Saff, Giza, Egypt, was selected (Farm 1). Due to limited access to other neighboring farms, another neighboring farm with 7000 Ross broiler chicks was used as a control farm for comparison purposes at the selling age (Farm 2). The main concerns, from a biosecurity point of view, included the lack of optimal distances between farms (the distance between the two farms is 0.35 km), the high frequency of IBV outbreaks at the region, and the lack of shelters and enclosure systems that may allow contact with wild birds.

### 2.3. Vaccination and Sampling Protocol

Thirty serum samples were collected from farm 1 at day-old age to assess maternally derived antibodies (MDA) against IBV, Infectious bursal disease (IBD), and avian influenza of both H9 and H5 subtypes (AI-H9 and AI-H5). The only difference in vaccination programs between farms 1 and 2 was the use of IB-VAR2^®^ vaccine at day-old age, then a booster dose using IB-H120 at day 16 in farm 1, while IB-H120 was used at both ages in farm 2; along with the IBV vaccine, other vaccinations were routinely performed in the studied flocks as shown in Table 1. To assess the IBV vaccine efficacy, thirty birds were bled each time and sera were collected from the vaccinated birds at 7, 14, 21, and 28 days of life to evaluate the IBV immune response as well as IBD, AI-H9, and AI-H5 responses. At 5 days post-vaccination and at each sampling point mentioned above, tracheal and kidney tissues samples from 10 randomly selected birds were subjected to RT-PCR for viral pathogen detection. In general, if any clinical problem was noticed at any time point, collected samples were subjected to IBV RT-PCR and viral isolation.

### 2.4. Vaccinated Flock Performance

The flock health status was monitored on a daily basis for any clinical problems and/or any mortality. The final body weights measured at selling age, the total feed intake, and the feed conversion ratio (food conversion rate (FCR) = feed intake/weight gain) were calculated. Mortality was recorded daily and cumulative mortality was obtained. Farm 1 was compared to farm 2 at the selling age in terms of feed total weight gain, conversion ratio, and cumulative mortalities.

### 2.5. Assessment Humoral Antibody Levels against Selected Viral Pathogens

Collected sera were tested using ProFLOK^®^ IBV Ab and ProFLOK^®^ IBD Ab (Symbiotic, Edison, NJ, USA) for IBV and IBD viruses, respectively, following the protocols recommended by the manufacturer. Haemagglutination inhibition (HI) test was carried out using IBV antigens (IB-H120 and IBV-VAR2) prepared at our laboratory as previously described by the OIE manual [15], using recent Egyptian strains as inactivated viruses. The Haemagglutination inhibition (HI) reactivity was determined using a 1% suspension of chicken red blood cells.

### 2.6. Viral Pathogens Real-Time RT-PCR Detection

Viral RNA was extracted from tracheal swabs using the Patho Gene-spinTM DNA/RNA Extraction Kit (iNtRON Biotech, Daejeon, Korea) according to the manufacturer’s instructions. The Quant one-step RT-PCR Tiangen Kit (Tiangen Inc., Beijing, China) was used to perform single-step rRT-PCR assays using specific oligonucleotide primers and probes for AI-H5 [16], AI-H9 [17], IBV [18], and velogenic NDV [19]. The HI test was carried out for AI-H9 and AI-H5 according OIE manual [20].

### 2.7. Statistical Analysis

The statistical analysis is done using a two-tail T-test with *p* value = 0.05, using SAS software version 9.4 [21].

## 3. Results

### 3.1. Vaccinated Flock’s Performance

The vaccinated flocks at farm 1 and 2 remained free of any clinical disease signs. However, in farm 2 (control farm), respiratory symptoms with increased mortalities were observed by 22 days of age. Comparison of the two flocks revealed that the cumulative mortality rate was 9% and 22% in farm 1 and 2, respectively. Also, farm 1’s total sold weight was 12,250 kg with a FCR of 1.7, while farm 2 yielded a total of 8150 kg with a FCR of 2.1 (Table 2).

### 3.2. Humoral Immune Responses Evaluation

**Figure 1:**
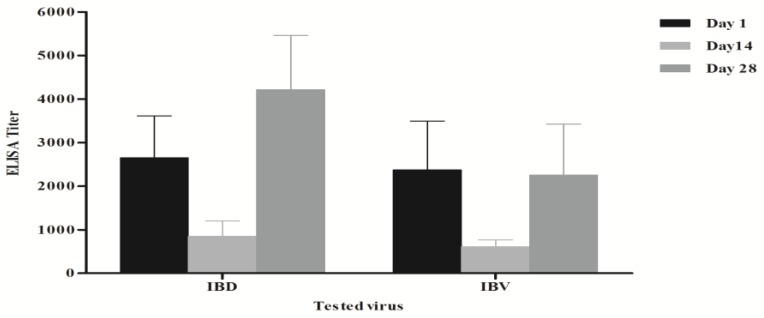
Mean enzyme-linked immunosorbent assay (ELISA) antibody titers against Infectious bronchitis virus (IBV) and Infectious bursal disease (IBD) at 0, 14, and 28 days of age, post-vaccination.

**Figure 2:**
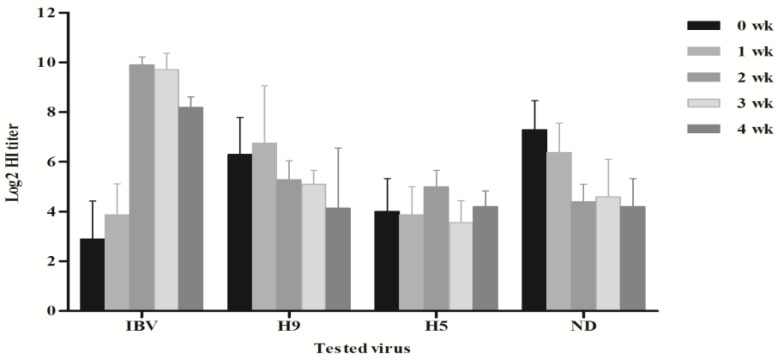
Weekly log2 Haemagglutination inhibition (HI) antibody titers against IBV, AI-H9, AI-H5, and Newcastle Disease virus (NDV) vaccines.

The mean maternally derived antibody levels (MDA) for IBV at day-old age was 2372 ± 1123 by enzyme-linked immunosorbent assay (ELISA). At 14-Day post vaccination (DPV), the ELISA titers dropped to 590 ± 155. By 12 days after the second IBV vaccination using the IB-H120 vaccine, the ELISA titers were elevated to reach 2253 ± 1175 (Figure 1). Weekly monitoring of antibody level titers in farm 1 had been done. The IBV HI antibody titers show that the MDA log2 HI antibody titer using the IB-VAR2 antigen was 2.9 ± 1.5; however, using the classical IBV antigen (IBV-H120) indicated that the MDA titers were 5.4 ± 1.3 log2 (data not shown). The HI titers using the homologues IB-VAR2 showed that the log2 HI antibody titers increased to 9.9 ± 0.3 log2 by two weeks post-vaccination and remained high (≥9 log2) till five weeks post-vaccination (Figure 2).

The immune responses against other vaccines showed that the IBD mean ELISA titers decreased from 2653 ± 965 to 848 ± 353 by two weeks of age as a result of MDA decay. As a result of IBD vaccine application, the IBD mean titers were elevated to 4210 ± 1248 at 28 days of age (Figure 1). The AI-H9, AI-H5, and NDV log2 HI antibody titer monitoring demonstrated that there were no adverse effects of the IB-VAR^®^ on the other vaccines administered to the flock. The lowest responses to the used vaccines were at three and four weeks of age for AI-H5 and AI-H9, respectively, and at five weeks of age for NDV (Figure 2).

### 3.3. Detection of Selected Viral Pathogens 

As shown in Table 3, with the exception of IBV, none of the tested viral pathogens were detected at any time point. The IBV virus detection rate was high at five days of age (i.e., after vaccination) and was mainly in the tracheal samples. The virus was not detected in the kidney by 14 days of age. The virus detection rate increased by 21 days of age in farm 1, and there was a respiratory disease problem in farm 2. Samples collected from both farms were subjected to virus isolation and IBV S1 gene partial sequencing. The results revealed that a wild-type virulent IBV variant 2 strain was implicated in this outbreak. The same virus strain was isolated from both farms (data not shown).

## 4. Discussion

IBV is a major cause of respiratory problems in broilers and alters egg production in breeders and layers in terms of quality and quantity. Recently, the Egyptian poultry industry has suffered from frequent outbreaks of respiratory diseases associated with variant II IBV strains [4,22]. The heterologous challenges, immunosuppression, concurrent infections, and inadequate biosecurity measures were found to be the main causes of vaccine failures [22,23,24].

With the aim to broaden the protective effectiveness of IBV vaccines, an inconsistently successful simultaneous vaccination of live classic Massachusetts and imported variant IBV vaccines has been attempted in commercial broiler chickens in Egypt. However, the efficacy still does not meet requirements, especially with the significant genetic and antigenic differences between the vaccine and field strains [22]. The use of genetically related strains as vaccines depended upon previous studies that confirmed successful protection using homologous IBV vaccine strain virus with the Chinese QX-like IBV [10,11] and Korean nephropathogenic IBV strains [12].

In this study, a newly developed live attenuated vaccine prepared from an Egy/VarII strain representing the GI-23 Middle East lineage (IB-VAR2^®^) was evaluated in a prime-boost IBV vaccination program in commercial broiler chickens under field conditions to monitor safety and efficacy. The laboratory evaluation of specific pathogen-free chicks in Biosafety Level-3 (BSL-3) isolators with ciliary activity evaluation and pathological lesions in the respiratory tract and kidneys were conducted in another trial [25].

The IB-VAR2^®^ vaccination at day-old age showed that the vaccine was tolerable in day-old commercial broiler chicks under field conditions with no adverse clinical manifestations through the study period. The final sold total weight and FCR of the vaccinated flock in farm 1 were better compared to those of the IB-H120-vaccinated flock from farm 2. The IBV virus detection, isolation, and identification confirmed the exposure of the two farms to a wild-type variant II IBV strain. The exposure to wild-type IBV had a great impact on farm 2 (IB-H120), with increased mortality in farm 2 considering the same biosecurity concerns in both farms.

The phenomenon of viral interference was previously reported between IBV and live NDV vaccine strains [26]. However, recent studies showed that the IBV interference with NDV replication was not reflected in a reduction in NDV immunity and that milder NDV vaccines were more susceptible to IBV interference [27,28]. To assure that the used IB-VAR2 vaccine has no effect on other viral vaccines’ humoral immune responses, the antibody levels against NDV, IBD, AI-H9, and AI-H5 vaccines were also monitored. No adverse effect of the IB-VAR2 vaccine on any of the tested viruses’ humoral immune responses was detected. The relatively low HI antibody titers of AI-H9 and AI-H5 recorded at two weeks of age are probably due to MDA decay; however, the titers were raised by two weeks of age due to the inactivated AI vaccine applications at days 7 and 9 for AI-H9 and AI-H5, respectively.

The IBV immune response was monitored by both ELISA and HI test. MDA titers of IBV were very low using the IB-VAR2 antigen as compared to those detected by the IB-M41 classical IBV antigen. These findings are consistent with previous research, indicating that the IBV HI test is strain-specific [29]. The maximum IBV ELISA antibody titers were detected at day 1 of age (i.e., MDA titers) and at 28 days of age (i.e., 12th day post-second IBV vaccination with H120). The observed low to moderate levels of IBV ELISA antibody titers at the 14th day of age in 60% of vaccinated birds were similar to other observations with both vaccine strains [30,31,32] and virulent IBV inoculation [33]. The high IBV HI antibody titers until the 35th day of age and the high antibody ELISA antibody titers at the 28th day of age were almost similar to the data reported previously with Massachusetts and 793B serotype IBV vaccines [34].

None of the targeted viral pathogens were detected by rRT-PCR at any time point. IBV rRT-PCR detection was mainly limited to the trachea, and no virus was detected in the kidneys by the 14th day of age. The very limited detection of IBV in the kidneys supports the safety of the vaccine as compared to its nephropathogenic wild-type parent virus. The limitation of the fact that the rRT-PCR assay cannot differentiate between vaccine and field IBV strains [18] is acknowledged in this study; however, the clinical performance of the vaccinated flock and low rates of IBV virus detection indicated that there was no need to go further regarding identification of the IBV virus.

## 5. Conclusions

In conclusion, this study indicated the safety and effectiveness of the newly developed live attenuated ME VAC IB-VAR2^®^ vaccine (representing the GI-23 Middle East lineage) in day-old commercial broiler chickens in highly endemic areas; also, the ME VAC IB-VAR2^®^ vaccine was better able to protect against the disease in an IBV-endemic study area. The vaccine does not interfere with the birds’ immune response to both live and inactivated vaccines of other viral pathogens. Moreover, it appears that the combination of ME VAC IB-VAR2^®^ and live classical IBV vaccines confers a protective immune response; however, protection studies are needed to confirm the ability of different vaccine combinations to protect chickens from both homologous and heterologous viral challenges.

## Figures and Tables

**Table 1:** Vaccination programs adopted in the studied commercial broiler flock. IBV: infectious bronchitis virus. AI: avian influenza.

Age	Vaccine		Type	Route
	Farm 1	Farm 2		
Day 1	IB-VAR2	IB-H120	Live IBV	Eye drop
Day 4	Newcastle Disease virus (NDV)		Killed NDV	Subcutaneous
	Newcastle Disease virus		Live NDV	Eye drop
Day 7	AI-H9N2		Killed H9N2	Subcutaneous
Day 9	AI-H5N1		Killed H5N3	Subcutaneous
Day 12	Infectious bursal disease (IBD)		Live IBD	Drinking water
Day 16	LaSota + IB H120		Live NDV and IBV	Eye drop

**Table 2:** Comparative flock performance data of farm 1 and 2.

Item	Farm 1 (IB Var2/H120)	Farm 2 (H120/H120)
Total no. in flock	7600	7000
Total no. of sold birds	6916 ^a^	5460 ^a^
Cumulative mortality	9.0%	22.0%
Total sold weight	12,250 ^b^ kg	8150 ^b^ kg
Average weight	1771 ^c^	1492 ^c^
FCR	1.7 ^d^	2.1 ^d^

^a^ The total sold live birds in farm 1 is statistically significantly higher than that of farm 2 with *p* < 0.05; ^b^ the total sold bird weight from farm 1 is statistically significantly higher than that of farm 2 with *p* < 0.05; ^c^ the geometric average individual bird weight at 35 days old from farm 2 is statistically significantly higher than that of farm 1 with *p* < 0.05; ^d^ the food conversion rate (FCR) of birds from farm 2 is statistically significantly higher than that of farm 1 with *p* < 0.05.

**Table 3:** Selected viral pathogen real-time RT-PCR detection in farm 1 and 2.

Farm	Age (Days)	Viral Pathogens				
		AI-H5	AI-H9	vNDV	IBV	
					Trachea	Kidneys
Farm 1 (IB-VAR2)	5	-	-	-	+(9/10)	+(2/10)
	7	-	-	-	+(2/10)	+(2/10)
	14	-	-	-	−(0/10)	−(0/10)
	21	-	-	-	+(6/10)	+(4/10)
	28	-	-	-	+(3/10)	NT
	35	-	-	-	+(3/10)	NT
Farm 2 (IB-H120)	22	-	-	-	+(8/15)	+(10/15)

vNDV (Velogenic Newcastle disease virus); NT: not tested.
